# Transcranial magnetic stimulation for diplopia in a patient with spinocerebellar ataxia type 6: a case report

**DOI:** 10.1186/s40673-018-0094-x

**Published:** 2018-11-20

**Authors:** Kentaro Kawamura, Seiji Etoh, Megumi Shimodozono

**Affiliations:** Department of Rehabilitation and Physical Medicine, Kagoshima University Graduate School of Medical and Dental Sciences, 8-35-1, Sakuragaoka, Kagoshima city, Kagoshima, 890-8520 Japan

**Keywords:** SCA6, Diplopia, Single pulse, TMS, Ataxia, Ophthalmoparesis, Nystagmus, Motor cortex, Cerebellum, Binocular fusion

## Abstract

**Background:**

In Patients with spinocerebellar ataxia type 6 (SCA6) are often treated by transcranial magnetic stimulation (TMS) over the motor cortex and cerebellum. However, few reports have examined effective therapeutic modalities for diplopia in SCA6 patients. In the current case, we applied single-pulse TMS over the motor cortex and cerebellum to improve ataxia, and observed an unexpected improvement of diplopia.

**Case presentation:**

A 62-year-old Japanese male with spinocerebellar ataxia type 6 (SCA6) was admitted to our hospital for exacerbation of ataxia. We administered single-pulse transcranial magnetic stimulation (TMS) over the hand motor area and the cerebellum with a circular coil to reduce ataxia. After the initiation of TMS, since diplopia unexpectedly improved, we started a quantitative assessment of diplopia by counting the number of fixation spots that he observed in his visual field. This assessment suggested that TMS had an immediate and cumulative effect on diplopia. We also delivered more localized stimulation only over the motor cortex with a Figure-8 coil, and diplopia improved immediately. Additionally, we administered a sham stimulation before the real stimulation over the motor cortex and the cerebellum. The sham stimulation improved diplopia, and greater improvement was observed with subsequent real stimulation. We also used a Hess chart examination and video recordings of binocular gross appearance to elucidate the changes in ocular movement objectively. However, these examinations did not reveal any obvious oculomotor changes.

**Conclusions:**

We applied single-pulse TMS to a SCA6 patient with diplopia, which improved without any adverse effects. TMS may have potential for the treatment of diplopia in SCA6 patients.

## Introduction

Spinocerebellar ataxia type 6 (SCA6) is an autosomal dominant neurodegenerative disease that is characterized by progressive symptoms such as ataxia, pyramidal and extrapyramidal disorder, dysphagia, sensory disturbances and oculomotor disorders including diplopia [1–3].

SCA6 patients are often treated by transcranial magnetic stimulation (TMS) over the motor cortex and cerebellum [4–6]. However, few reports have examined effective therapeutic modalities for diplopia in SCA6 patients. In the current case, we applied single-pulse TMS over the motor cortex and cerebellum to improve ataxia, and observed an unexpected improvement of diplopia. We tried to verify the ocular changes that could induce such improvement, and speculate on the underlying mechanisms.

## Patients and methods

### Case report

**Fig. 1:**
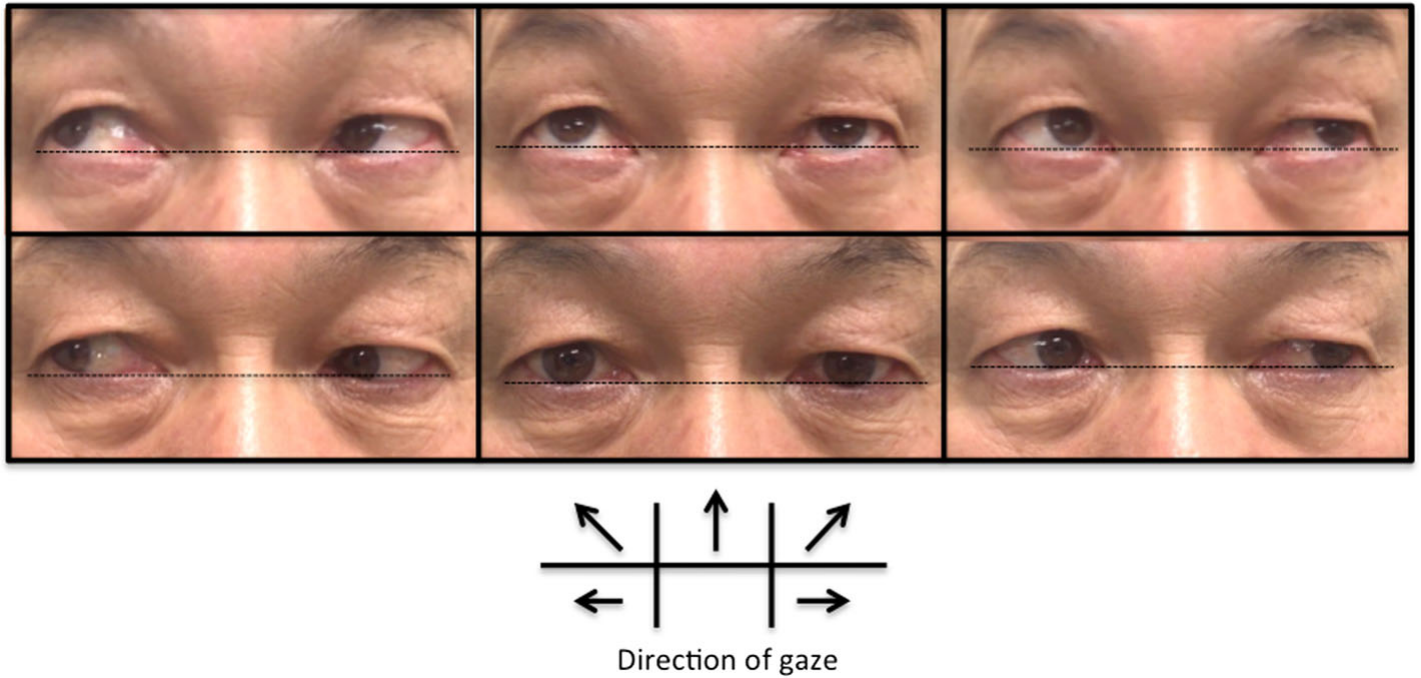
Extra-ocular movement just before the 8th TMS in the 1st course. The patient exhibited vertical misalignment for the upper and upper lateral gaze, especially the upper-right gaze

A 62-year-old Japanese male with SCA6 was admitted to our hospital due to exacerbation of ataxia. He developed diplopia and body sway in his 30s. These symptoms gradually progressed, and gait disturbance and dysarthria appeared in his 50s. At age 61 years, he was genetically diagnosed as SCA6. He could walk by himself with bilateral Lofstrand crutches, but had recently begun to fall frequently. He had a family history of SCA6 in his mother and 2 siblings. None of them could walk by themselves and were using a wheelchair. Clinical examination revealed truncal and limb ataxia, dysarthria, horizontal gaze-evoked nystagmus, and slight vertical misalignment (slight restriction of upper ocular movement of the left eyeball) in a neurological confrontation test. The misalignment was clearer for the upper-right gaze (Fig. 1). He had no cognitive dysfunction, his fundus was normal and he had no low vision.

### TMS protocol

In addition to physical therapy, we started TMS therapy to improve his ataxia. TMS over the motor cortex and the cerebellum was administered once a day for one session, and for five sessions a week for 2 weeks (10 sessions). This therapy was performed for 2 courses, separated by a rest period of about 2 weeks (total 20 sessions). We measured the resting motor threshold (RMT) by stimulating the hand area of the left primary motor cortex with a Magstim 200 (Magstim Co. Ltd., Whitland, UK) and a 14 cm circular coil. Next, we recorded the motor evoked potentials at the right abductor pollicis brevis muscle while the hands were relaxed. The RMT was defined as the minimum stimulation intensity necessary to induce a motor evoked potential exceeding 50 μV for more than half of the stimulations. The coil was placed tangentially over the scalp and centered near the Cz (for motor cortex stimulation) and inion (for cerebellar stimulation). The stimulator output was adjusted at 100% of RMT for the motor cortex and at 50% of RMT for the cerebellum. For the motor cortex stimulation, 20 single pulses (about 0.3 Hz) were delivered with current flowing counter-clockwise, followed by 20 clockwise pulses. Subsequently, 10 single pulses (about 0.5 Hz) were delivered over the cerebellum by the same procedure. Thus, in each daily session we delivered 40 pulses over the motor cortex followed by 20 pulses over the cerebellum.

Written informed consent was obtained from the patient before starting TMS therapy, which was approved by the local ethics committee (27–203).

### Diplopia assessment

Initially, we paid attention to and started to assess ataxia, but after the initiation of TMS, when we noted that diplopia had unexpectedly improved, we also began quantitative assessments of the area of diplopia during the latter half of the first course.

**Fig. 2:**
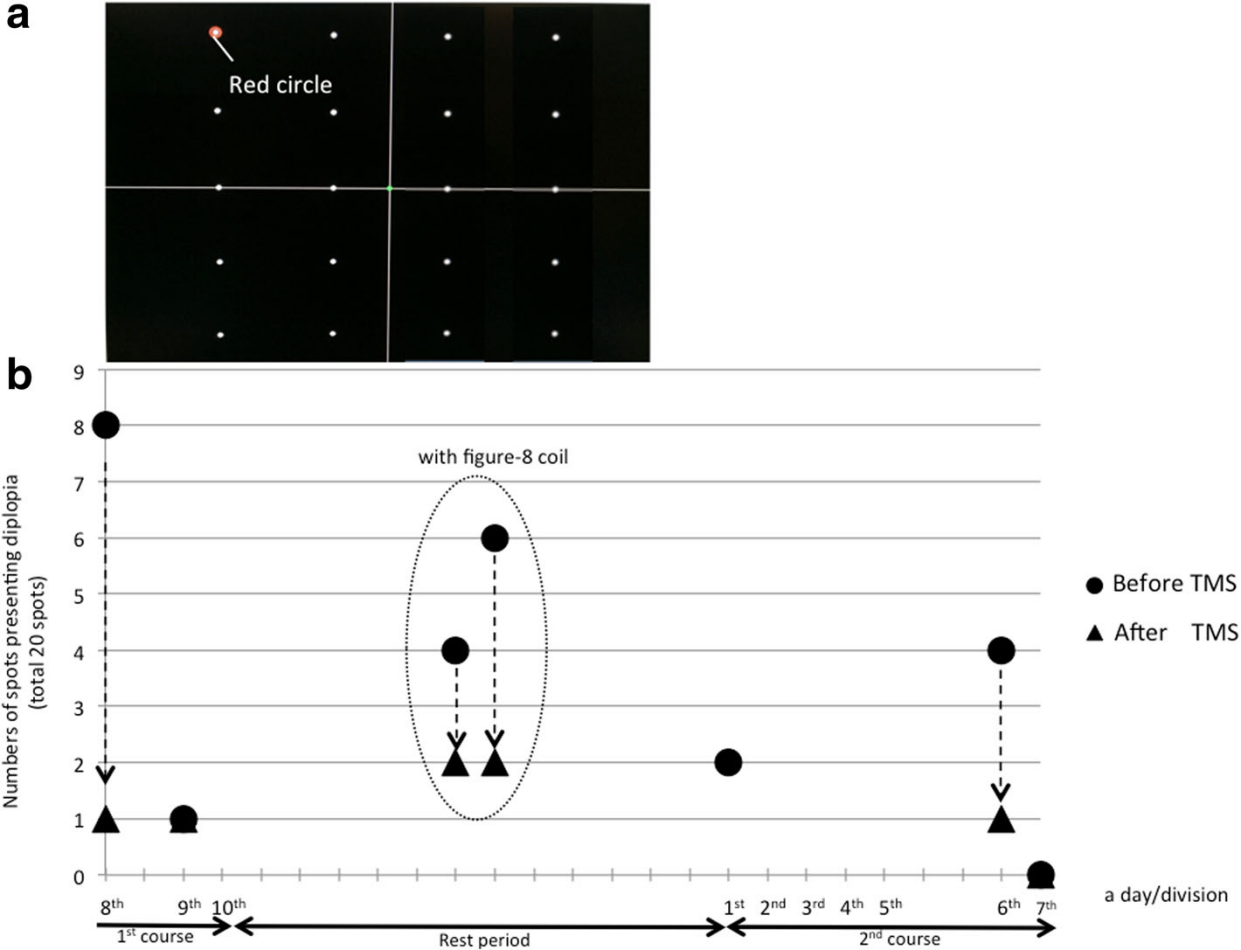
**a**: Initial arrangement of the spots used to detect locations presenting diplopia on a 20-in. PC monitor, **b**: Changes in the number of “diplopia-spots”. We started the assessment from immediately before and after the 8th TMS session, and thus did not assess all TMS sessions. We performed assessments in the 8th and 9th sessions in the 1st course and the 1st, 6th, 7th, 9^th^and 10th sessions in the 2nd course. The 8th, 9th and 10th sessions in the 2nd course are shown in Fig. 3. Dotted arrows show the immediate change in diplopia-spots with TMS. On the 7th intervention in the 2nd course, diplopia disappeared from the evaluation range, and therefore extended the range of assessment. On two days during the rest period between the 1st and 2nd courses, diplopia improved immediately after localized stimulation over the motor cortex with a Figure-8 coil (In a dotted oval)

We referred to a published protocol for quantitative assessment of the area of diplopia [7, 8]. Since we did not have a Goldmann perimeter, we used a PC monitor instead. We placed 20 fixation spots on a 20-in. PC monitor (Fig. 2a). The patient’s head was fixed with a jaw-receiving stand. We moved a red circle that surrounded a spot and instructed the patient to gaze at each surrounded spot one by one. For each spot, the patient stated whether he had diplopia, and we counted the number of spots presenting diplopia; i.e., diplopia-spots. The distance between the patient and monitor was 56 cm. The maximum angle of fixation was 12° laterally from the center and the vertical angle was from 2° above to 21° below his viewing height.

## Result

**Fig. 3:**
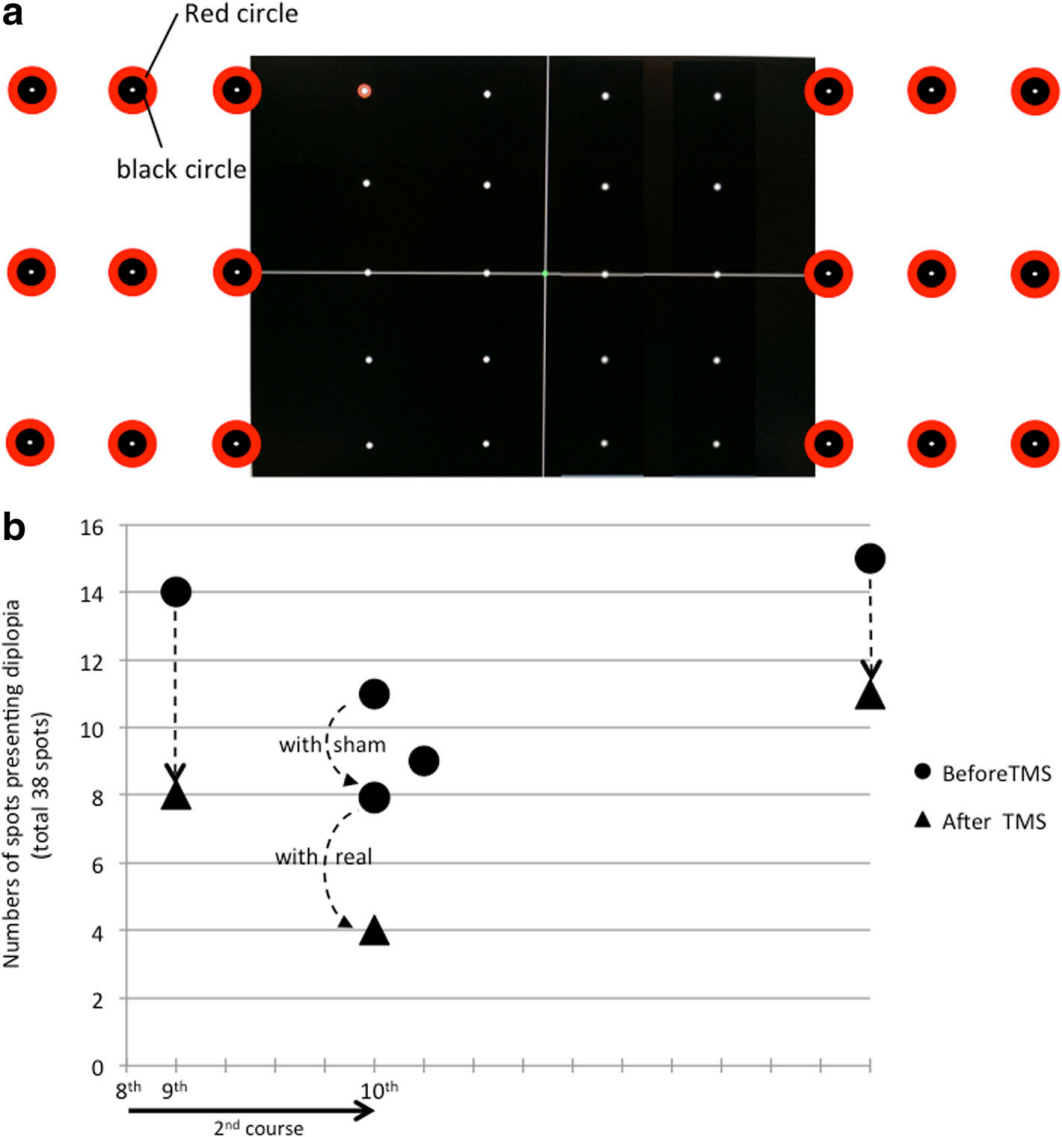
**a**: Arrangement of 18 spots added beyond the PC monitor for detecting positions presenting diplopia. **b**: Change in numbers of “diplopia-spots” among the 18 spots added beyond the PC monitor. On the day after the 10th TMS, only the assessment of diplopia-spots was performed (no TMS). Ten days after TMS therapy was complete, the number of diplopia-spots within the extended assessment range increased, but no spots appeared within the range of the initial assessment (on the monitor) and TMS had an immediate effect

The changes in the number of diplopia-spots are shown in Fig. 2b. At the 8th TMS session in the 1st course, there were eight diplopia-spots before TMS therapy, and only one (in the upper-right of his visual field) immediately after TMS. The patient reported a remarkable improvement of diplopia immediately after stimulation of the motor cortex and no further improvement after subsequent stimulation over the cerebellum. Since we supposed that stimulation over the motor cortex was more effective, we also delivered more localized stimulation only over the right motor cortex with a Figure-8 coil (40 single pulses, intensity 100% of RMT) on two days between the 1st and 2nd courses. On both days, immediately after this localized stimulation, diplopia remained only at spots in the upper-right of the visual field. At the 7th TMS in the 2nd course, diplopia-spots disappeared from within the assessment range (on the monitor). We added nine fixation spots to each visual field beyond the monitor to extend the assessment range. Each added fixation spot consisted of a φ5mm black circle surrounded by a φ1.5 cm red circle (Fig. 3a). The fixation angle was increased laterally to 36°. Figure 3b shows the number of diplopia-spots and the time course after the range was increased. In this range, the number of diplopia-spots again decreased immediately after TMS.

In the last TMS session, sham stimulation was delivered over the motor cortex and the cerebellum before real stimulation. A sham Figure-8 coil was placed at the same point where the circular coil (real stimulation) was centered. The sham stimulation was performed at the same frequency, and included clicking sounds that mimicked those during real stimulation. The number of diplopia-spots decreased with sham stimulation, and decreased even further with real stimulation (Fig. 3b).

At 10 days after TMS therapy was complete, diplopia-spots increased within the extended assessment range, but none appeared in the initial assessment range (on the monitor), and TMS had an immediate effect. Throughout both courses of treatment, diplopia tended to remain in the upper-right of the visual field.

**Fig. 4:**
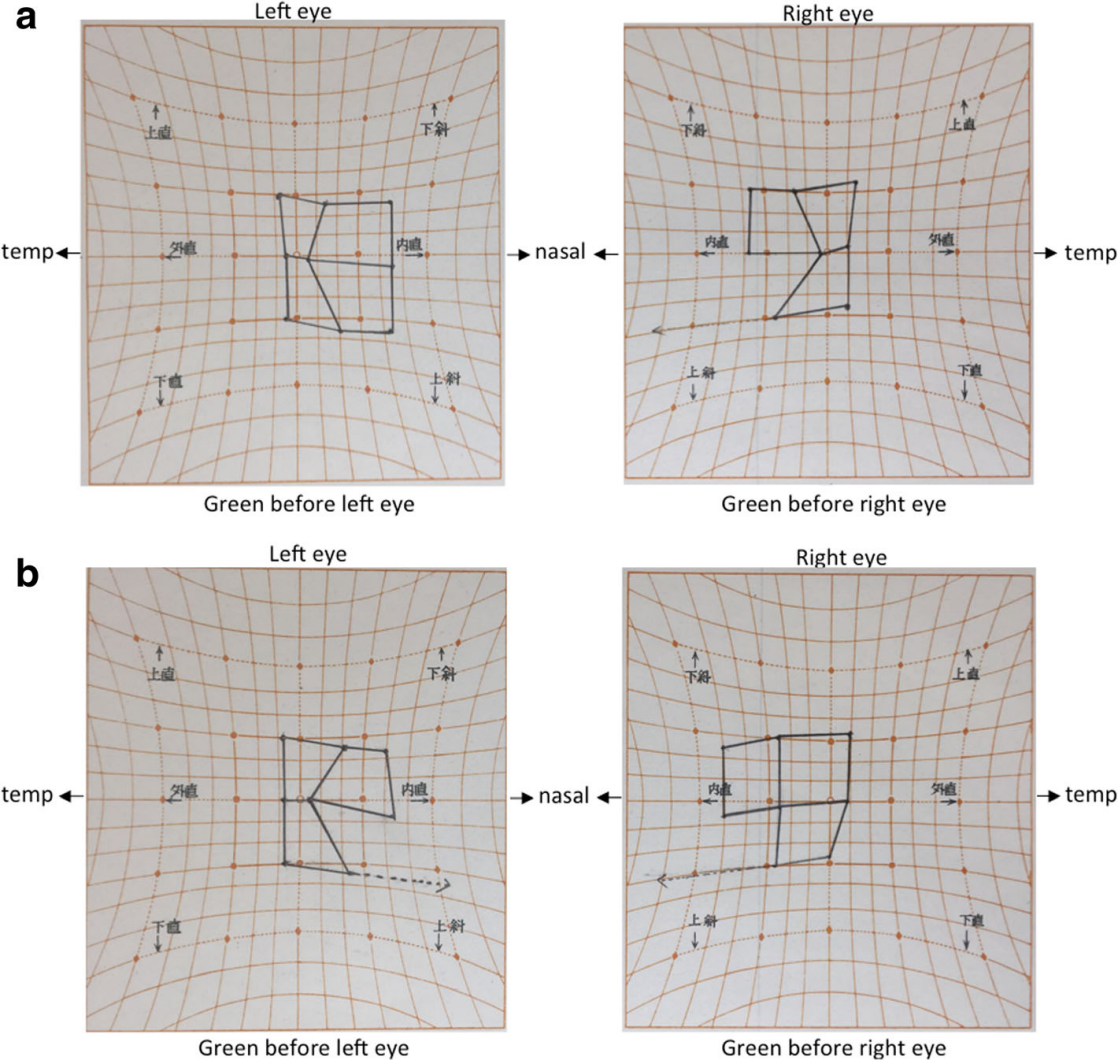
**a**: Hess charts at the beginning of the diplopia assessment. **b**: Hess charts after all courses were complete

**Fig. 5:**
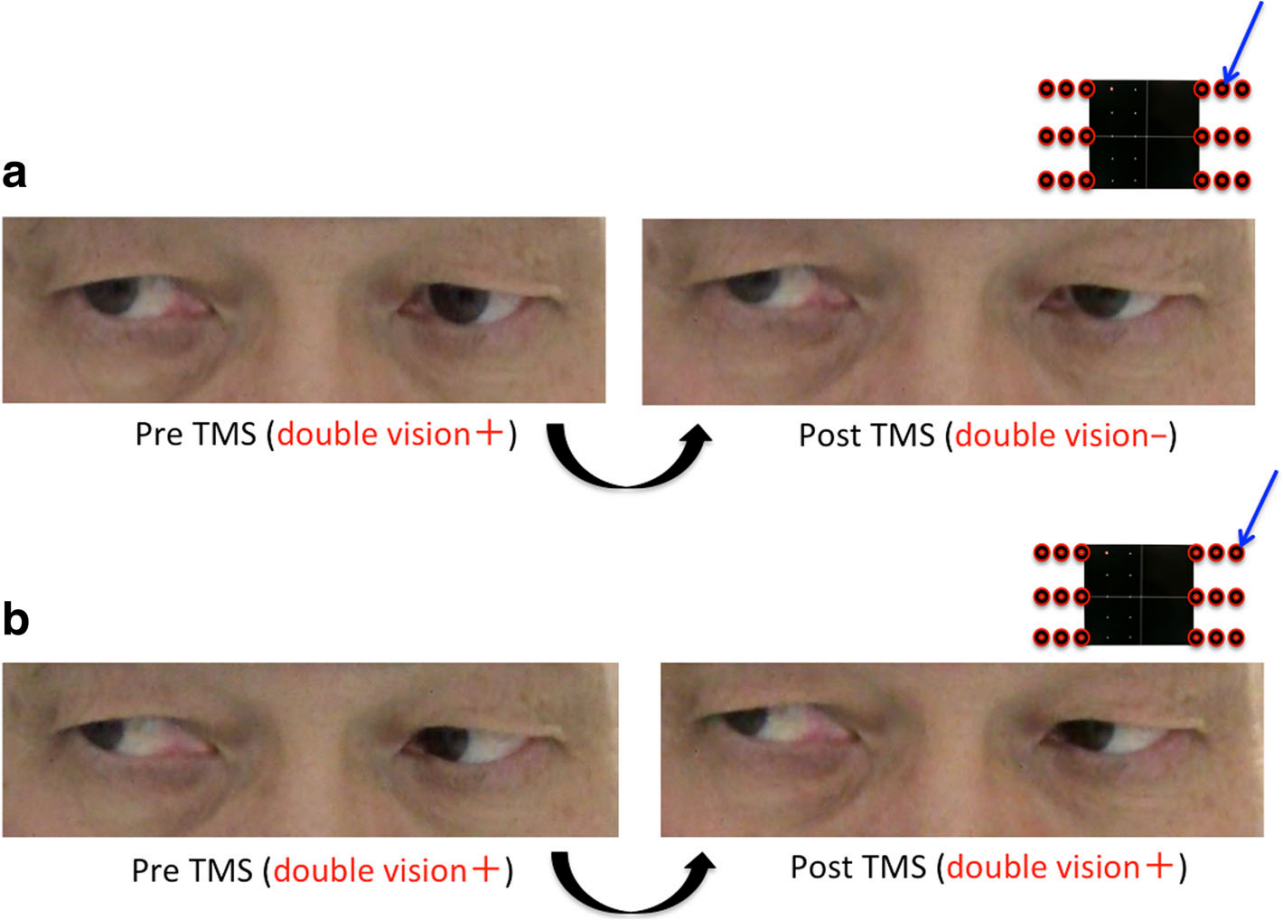
**a**: Comparison of oculomotor movement with video recordings at the upper-right spot where diplopia improved. **b:** Comparison of oculomotor movement with video recordings at the upper-right spot where diplopia did not improve

We used a Hess chart and video recordings (with a camcorder) of the binocular gross appearance to objectively elucidate the changes in ocular movement. With the Hess charts, an unknown bilateral abduction disorder was suspected at the beginning of the diplopia assessment, and no changes in Hess charts were observed after both courses were complete (Fig. 4a & 4b). In the video recordings, we compared oculomotor movement before and after TMS at spots where diplopia disappeared and where diplopia did not disappear after the TMS. However, we did not detect obvious oculomotor differences between before and after TMS (Fig. 5a & 5b).

**Fig. 6:**
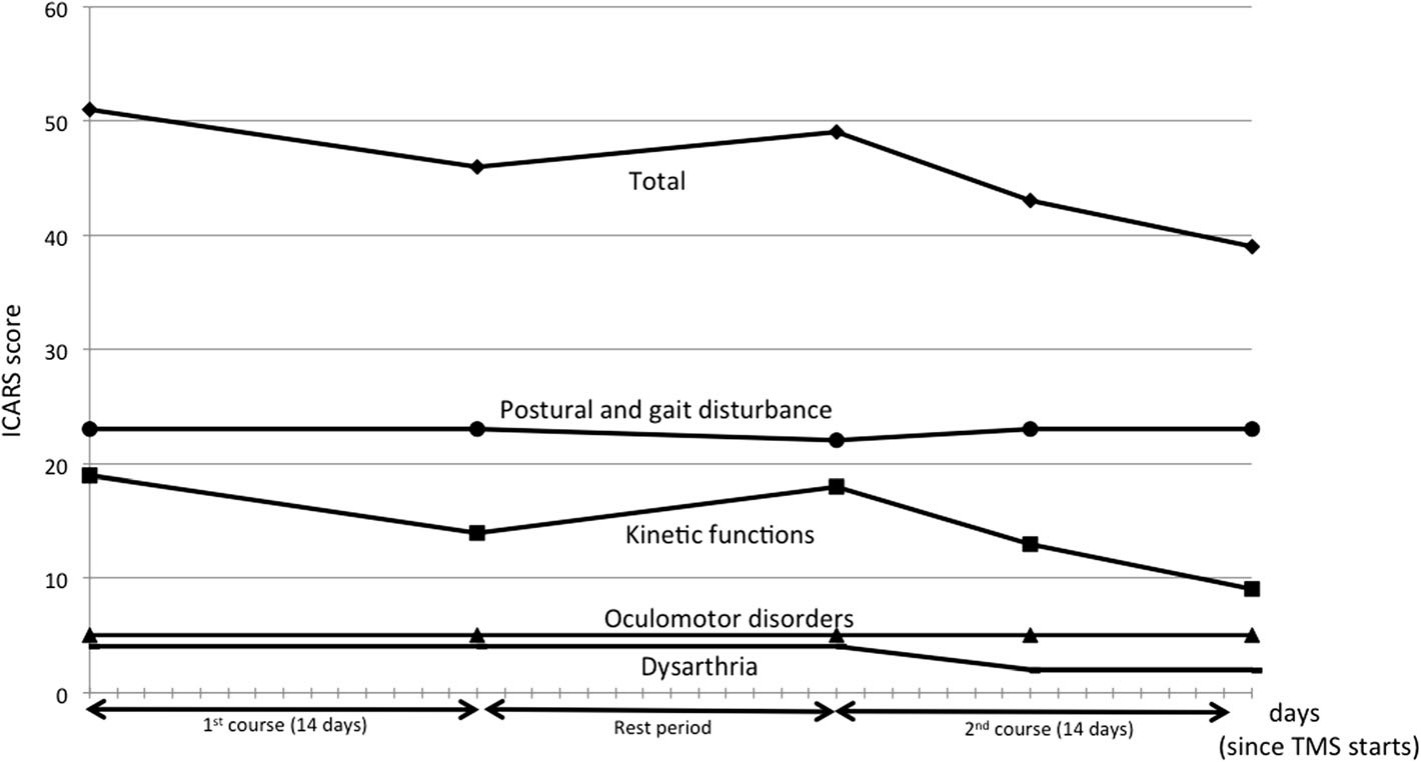
Changes in the score on the International Cooperative Ataxia Rating Scale (ICARS). Total scores (Full scores, 100) consist of Postural and gait disturbance (full scores, 34), Kinetic functions (full scores, 52), Oculomotor disorders (full scores, 8) and Dysarthria (full scores, 6). As the ICARS score increases, the symptoms become more severe. In the current case, improvement of kinetic functions seemed to be related to TMS therapy

Furthermore, we assessed ataxia using the International Cooperative Ataxia Rating Scale (ICARS), and the results showed that limb ataxia gradually improved (Fig. 6).

## Discussion

In this case, TMS over the motor cortex and the cerebellum had an immediate and cumulative effect not just for ataxia, but also for diplopia. To the best of our knowledge, this is the first report in which TMS improved diplopia in a SCA6 patient.

Especially, diplopia improved immediately after localized, one-side (right side), stimulation (with a Figure-8 coil) over the motor cortex. In addition, the intensity of the stimulation over the cerebellum was comparatively weak compared to previous reports [4–6] and the patient was conscious of the improvement immediately after stimulation of the motor cortex. Therefore, we considered that the improvement was mainly due to stimulation over the motor cortex.

In this case, since vertical misalignment (especially for the upper-right gaze) and the upper-right diplopia spot tended to remain after stimulation, we considered that this ophthalmoparesis was the main cause of diplopia. To detect changes associated with this ophthalmoparesis objectively, we used a Hess chart examination and video recordings before and after TMS. However, we could not identify any significant changes. It is possible that the oculomotor changes were too small to be identified with these instruments or the improvement of diplopia may have been due to some cause other than oculomotor changes.

Several possible mechanisms could account for this diplopia-improvement with TMS. First, we can speculate that the excitability of the cerebellum changed with TMS over the motor cortex, which might induce oculomotor changes. Previous studies have shown that there are neural networks between the cerebral cortex and the cerebellum [9, 10], and that TMS over the motor cortex increases bilateral cerebellar blood flow [11, 12]. Increased blood flow may be associated with the excitability of the cerebellum. Since the cerebellum has efferent fibers to the vestibular nucleus (part of the neural integrator) which is connected to the oculomotor nucleus [13], the increased cerebellar excitability might regulate these connections and lead to some oculomotor improvement. The incidence of ophthalmoparesis in SCA6 patients has been reported to range from less than 10% to about 50% [1, 14]. In a prospective multicenter study, the frequency of ophthalmoparesis in SCA6 patients with diplopia was lower than those in other SCAs; 43% of SCA6 patients had diplopia, and 4 and 14% of these patients showed horizontal and vertical ophthalmoparesis, respectively [14]. These results suggest that, in addition to ophthalmoparesis, diplopia may be related to other oculomotor disorders, particularly in SCA6 patients. In SCA6 patients, gaze-evoked nystagmus (GEN), dysmetric saccade (hyper > hypo), smooth pursuit, square-wave jerks, and other forms of nystagmus (rebound, periodic alternating, and downbeat nystagmus) have been reported [1–3, 9, 14]. Among these disorders, our patient had GEN, hypermetric saccade, smooth pursuit disorder, and rebound nystagmus. Since diplopia appeared with lateral eye fixation in this case, GEN was a plausible cause. Since nystagmus could induce diplopia [15] as well as oscillopsia, the improvement of GEN might lead to the improvement of diplopia. However, we could not detect a change in GEN with our video recordings. Although we did not detect changes in GEN objectively with our limited instruments, we might have been able to detect changes if we had assessed oculomotor movement in detail with the use of electrooculograms, nystagmography, 3D eye-tracking, etc.

Second, the frontal eye field (FEF) might have been stimulated in this patient. The FEF projects to the superior colliculus in the midbrain, where ocular movement signals are assumed to be integrated [16]. Since the FEF is believed to be relatively close to the primary motor cortex [17], the stimulus might have spread there and indirectly affected ocular movement and gazes. Interestingly, TMS over the FEF has been reported to facilitate saccades and visual cognition [16, 18]. It is possible that these factors might have contributed to the improvement of diplopia.

Third, previous studies have shown that a complicated brain network (including the frontal, parietal, occipital, and temporal lobules) is related to binocular fusion [19]. The stimulus could have affected this network, and the change in binocular fusion (especially in sensory fusion) might have induced the improvement in diplopia. For a more detailed assessment of fusion, the Worth Four Dot test and Titmus stereo test should have been done.

In the current case, the cause of diplopia was not detected with our limited instruments, and the improvement in diplopia was based on a subjective and quantitative evaluation. It would have been better if we could clarify the cause of diplopia and demonstrate that diplopia had indeed improved by an objective and qualitative evaluation.

## Conclusion

We applied single-pulse TMS to a SCA6 patient with diplopia, which improved without any adverse effects. TMS may have potential as a therapeutic technique for diplopia in SCA6 patients. Further studies are needed to evaluate the effect of TMS for diplopia in SCA6 patients.

## Abbreviations

FEF: Frontal eye field
GEN: Gaze-evoked nystagmus
ICARS: International Cooperative Ataxia Rating Scale
RMT: Resting motor threshold
SCA6: Spinocerebellar ataxia type 6
TMS: Transcranial magnetic stimulation
